# Self-certification: A novel method for increasing sharing discernment on social media

**DOI:** 10.1371/journal.pone.0303025

**Published:** 2024-06-11

**Authors:** Piers Douglas Lionel Howe, Andrew Perfors, Keith J. Ransom, Bradley Walker, Nicolas Fay, Yoshi Kashima, Morgan Saletta, Sihan Dong

**Affiliations:** 1 School of Psychological Sciences, University of Melbourne, Melbourne, VIC, Australia; 2 School of Psychological Science, University of Western Australia, Perth, WA, Australia; 3 School of Electrical Engineering, Computing and Mathematical Sciences, Curtin University, Perth, WA, Australia; 4 Hunt Laboratory, University of Melbourne, Melbourne, VIC, Australia; University of Potsdam: Universitat Potsdam, GERMANY

## Abstract

The proliferation of misinformation on social media platforms has given rise to growing demands for effective intervention strategies that increase sharing discernment (i.e. increase the difference in the probability of sharing true posts relative to the probability of sharing false posts). One suggested method is to encourage users to deliberate on the veracity of the information prior to sharing. However, this strategy is undermined by individuals’ propensity to share posts they acknowledge as false. In our study, across three experiments, in a simulated social media environment, participants were shown social media posts and asked whether they wished to share them and, sometimes, whether they believed the posts to be truthful. We observe that requiring users to verify their belief in a news post’s truthfulness before sharing it markedly curtails the dissemination of false information. Thus, requiring self-certification increased sharing discernment. Importantly, requiring self-certification didn’t hinder users from sharing content they genuinely believed to be true because participants were allowed to share any posts that they indicated were true. We propose self-certification as a method that substantially curbs the spread of misleading content on social media without infringing upon the principle of free speech.

## Introduction

Highly misleading and sometimes entirely fabricated news stories are being widely circulated on social media [1, 2]. These stories are often related to significant events and may encourage undesirable reactions to these events such as the January 6 Unites States Capitol Attack [3] and COVID-19 vaccine refusal [4]. Consequently, there are increasing calls for social media companies to do more to reduce the spread of misinformation on their platforms [5], and a majority of U.S. adults believe that tech companies should take such steps to do this even if this would result in losing some freedom to access and publish online content [6–8].

Several approaches have been developed to encourage users to be more discerning when sharing information on social media [9, 10]. These include media literacy training [11], fact-checking [12], debunking [13], inoculations [14] and source credibility warnings [15]. Particularly pertinent to this study are accuracy prompts, designed to nudge users into evaluating the truthfulness of the information they intend to share. This approach capitalises on the fact that a large majority of social media users report that it is important to share only accurate information [1]. Pennycook et al. suggested that users share false information despite this belief because they do not consider accuracy when sharing [1]. This suggests that reminders (i.e. nudges) to consider accuracy should increase sharing discernment (please see [1] for further discussion). Despite some impressive initial findings, the effectiveness of such nudges has been disputed [16, 17]. In part to address these concerns, a meta-analysis was conducted [18]. Pennycook et al. defined sharing discernment as the difference in probability for sharing accurate versus inaccurate information (i.e. if a participant has an 80% probability of sharing true social media posts but only a 20% probability of sharing false social media posts their sharing discernment is 60%) [1]. Pennycook et al. found that that accuracy prompts reliably improved sharing discernment, but the effectiveness of this nudge was relatively small and varied across studies. One reason why accuracy prompts may not be particularly effective is that some users appear to share news posts even when they don’t believe they are accurate (e.g. study 6 of [1]). This suggests that accuracy is not the only factor to influence sharing decisions. This makes some sense: people might also share to signal their identity, to gain followers or “likes”, to influence other people, for entertainment, or because the post would be interesting if it were true [19]. Nevertheless, the act of sharing might still signal to the receiver that the shared item is true, so still spread misinformation. Here we investigate a novel intervention, which builds upon the accuracy prompt work we describe above [1]. We call this intervention “self-certification” because users are allowed to share only those posts that they have indicated are true. (In a previous publication we referred to this technique as “self-censorship” [20] but now believe that “self-certification” is a better term.) To be clear, there is nothing preventing the user from lying and certifying a post they believe is false as true so that they are allowed to share it. Nevertheless, we posit that most users will not lie, so this intervention will be more effective than accuracy prompts alone as users will not be allowed to share those posts that they have indicated as false.

## Experiment 1: Comparing self-certification to accuracy prompts

The primary purpose of the first experiment was to compare the effectiveness of self-certification to accuracy prompts for increasing sharing discernment in a simulated social media experiment. We also included a baseline condition that didn’t contain any interventions. Participants were shown twenty social media posts, one at a time. For each social media post, participants were asked to decide whether to share it. There were three conditions.

In the baseline condition, there were no accuracy prompts and no self-certification. In the accuracy prompt condition, participants assessed the accuracy of each news post immediately before deciding whether to share it. Pennycook et al. showed that this type of accuracy prompt is effective [1]. In the self-certification condition, participants were informed in advance that they would not be able to share any news posts that they indicated were false. They then rated the accuracy of each news post. Only if they indicated that the post was true were they then asked whether they wished to share it.

Based on the findings of Pennycook et al. [1], we predicted greater sharing discernment in both the accuracy prompt and self-certification conditions as these contained a reminder to consider accuracy that was absent in the baseline condition. As previously reported by Pennycook et al., we predicted that participants in the accuracy prompt condition would attempt to share posts that they had labelled as false. Since participants would be prevented from doing this in the self-certification condition, we predicted that less false information would be shared in the self-certification condition, leading to greater sharing discernment.

### Method

#### Participants

Participants were recruited via Mechanical Turk as this has been shown to be an efficient way of recruiting a diverse sample of high quality participants, providing appropriate screening measures are employed [21, 22]. The study was only open to the subset of Mechanical Turk workers who were located in the USA, had previously passed a test designed to assess English proficiency and to screen out bots. The study took approximately 5 minutes to complete and participants were compensated US$1 for their time. We excluded only those participants who didn’t finish the experiment. Participants were randomly assigned to one of the three conditions: 46 participants were assigned to the baseline condition, 53 to the accuracy prompt condition, and 50 to the self-certification condition. Out of 149 participants, 53 participants self-identified as female and 93 as male. The remaining participants self-identified as non-binary or “other”. The mean age was 39.8 years old (SD = 11.5 years). 28 participants self-identified as Republicans, 81 as Democrats, 36 as independents and four as “other”. All participants gave informed consent, and the study was approved by the University of Melbourne Human Ethics Advisory Group (ID: 23317).

#### Materials

**Fig 1:**
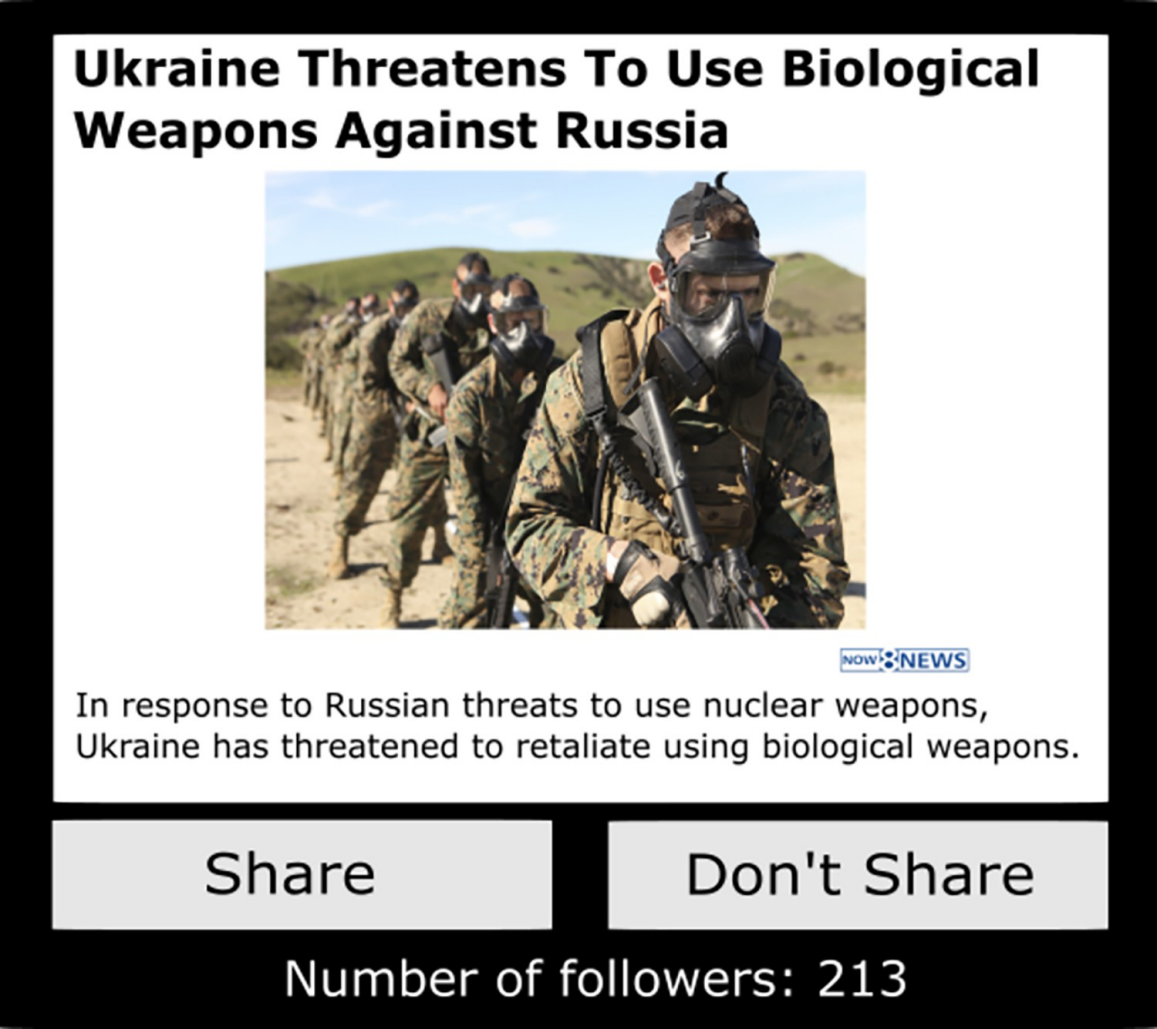
An example of a false social media post. Note that in the above image the photographs was replaced by a copyright-free photograph depicting similar content https://www.rawpixel.com/image/8742382/photo-image-public-domain-people.

Each participant was shown 20 simulated social media news posts (Fig 1). All the posts focused on the Russo-Ukrainian War. Half were true and half were false. The true posts were sourced from three reliable news sources: The New York Times, the BBC, and The Wall Street Journal, and were additionally checked to be true at the time the study was run. We purposely chose posts that were unlikely to be highly familiar to our participants and didn’t refer to well-known major events, otherwise their veracity would have been obvious. We also tried to choose posts that were not dated. Finally, we selected from the three sources approximately equally. Following the advice of Pennycook et al. [23], we selected the false news posts from a fact-checking website (Snopes.com) to ensure that we were using news posts that had previously gone viral. This means that our participants may have seen versions of some of these news posts before. To avoid ambiguity, we selected only posts that Snopes.com had unambiguously rated as “False”, ignoring posts rated as “Mostly False” or “Mixture”. This ensured that we utilised only those posts with a demonstrated ability to go viral, as these are the type of false posts that one needs to especially guard against. As with the true posts, we avoided any that seemed dated or that referred to well-known events. We checked that the false posts were still false at the time the study was run (13–14 January, 2023).

We put all the posts into the same format so that the format itself didn’t give any clues as to the veracity of the post. This format was similar to the format adopted by Facebook news posts except for three changes. First, we gave each post a title as we felt that this made it easier for participants to rapidly assess what the post was about and made the posts more engaging. Second, we used each news source’s actual logo to make the alleged source of the post easier to recognise. Conversely, Facebook typically lists each news source in dark grey all-caps and as a single word, which we found made it harder to identify the source. Finally, to improve contrast, we utilised a white background (as opposed to Facebook’s light grey background) and did not force each post’s photograph to span the page, to avoid distorting it. While these changes meant that our posts did not have the exact same format as Facebook posts, we felt that this was an acceptable compromise as we are interested in the propagation of misinformation on social media in general, not specifically on Facebook. We therefore felt it was important to make the posts as easy-to-read and as engaging as possible.

Each post comprised a title, a photograph, a source (located directly underneath the photograph) and a lede sentence (or two). Note, that our posts did not link to the full article (as a news post on Facebook would) as we would have no way of determining how much of that article participants would read. However, most people on social media typically do not follow the link the full article anyway [24].

Each post had a designated source, but the stated source wasn’t always the actual source of the post. For the posts that were actually true, the stated source was always one of the previously identified reliable sources. For the posts that were actually false, the stated source was randomly selected from one of the previously identified unreliable sources. All sources were used approximately equally often. Below each post two buttons were shown, but their exact nature depended on the condition. Below these buttons was a message indicating how many followers the participant currently had.

#### Procedure

The experiment was run using Psytoolkit [25, 26]. Participants were first asked their age, gender and the political party with which they most strongly identified. In all conditions, they were informed that they would be participating in a simulated social media experiment and that their task was to gain as many followers as possible. They were instructed that they could gain new followers by sharing social media news posts that their followers wanted to see. They were instructed that each news post would contain a photograph and that the source of each news post would be indicated directly under the photograph. They were informed that Now8News, The Daily Buzz, and the World News Daily Report often publish news that is not true whereas The New York Times, the BBC, and The Wall Street Journal almost never publish news that is not true. The first three (i.e. the unreliable) news sources were chosen as they often publish false news and have no consistent political bias [20]. The latter three (i.e. the reliable) news sources were chosen as they are reputable, have neutral news coverage and represent a range of political opinions, with left-leaning, central and right-leaning editorial coverage respectively. The participant’s understanding of the instructions was then tested via multiple choice questions and any misunderstandings corrected. Participants were then randomly assigned to one of the three conditions.

We employed a 2 x 3 design crossing post veracity with condition. In the baseline condition, there were no accuracy prompts nor any self-certification. Participants saw twenty social media posts, one at a time. The posts attributed to the three reliable sources were always true whereas those attributed to the three unreliable sources were always false. Every post they shared, regardless of whether it was true or false, gained the participant a random number of new followers, drawn from a normal distribution with a mean of 100 and a standard deviation of 20. We purposely choose this number to be the same for both true and false news posts to avoid biasing participants to share one type over the other.

The accuracy prompt condition was identical to the baseline condition except that participants were first required to indicate whether a post was true or false before being asked whether they wished to share it. The self-certification condition was identical to the accuracy prompt condition except that participants were told that they would only be allowed to share those social media posts that they first indicated were true. Consistent with this, participants were not asked whether they wish to share any social media posts they indicated were false. In all conditions, after the participant had viewed the 20 news posts, the experiment finished, they were debriefed and invited to leave any comments they had. The stimuli, raw data and analysis code are available at OSF: https://osf.io/pxma8/?view_only=d74e0d4d1d0e47f9adc061e9ddfd37fa

### Results

**Fig 2:**
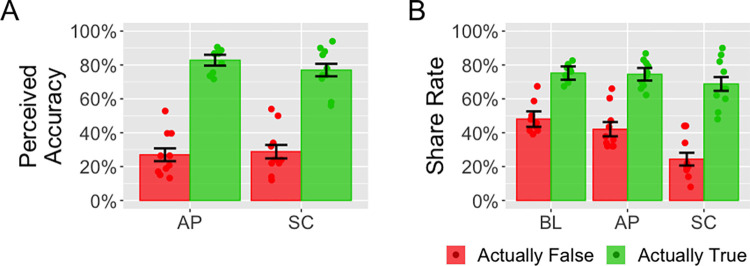
Results from Experiment 1. Subplot A shows the perceived accuracy, defined as the proportion of participants who indicated that each news post was true, for the accuracy prompt (AP) and self-certification (SC) conditions. Subplot B shows the share rate for the baseline (BL), AP and SC conditions. Each dot shows the data averaged over a single social media post. The bars show the averages over all social media posts that were actually false (red) or actually true (green).

The data were analysed using the lme4 software package [27] in R [28]. Linear mixed effects models were used to predict each participant’s response to each social media post. Specifically, these models predicted whether each participant thought each social media post was true (perceived accuracy model) and whether they wished to share it (sharing model). Although both these dependent variables are binary, we used linear models because our goal was to obtain unbiased estimates of the causal effects of our predictor variables [29]. For both the accuracy model and the sharing model, the predictors were *veracity* and *condition*, with random intercepts for *participant* and *social media post*. The predictor *veracity* denoted whether the social media post was actually true as opposed to how the participant perceived it. Adopting the terminology of [1], we defined sharing discernment as the difference in probability for sharing actually-true versus actually-false news posts. Thus, if the interaction between veracity and condition was significant, this would show that sharing discernment varied across conditions. Thus, when testing whether sharing discernment varied across conditions, we added the predictor veracity*condition to the sharing model. The data is summarised in Fig 2. (The preliminary data and analysis for this experiment was published in the conference proceeding for the 2023 Cognitive Science Conference [20]).

*Perceived accuracy* is the percentage of posts that were perceived to be true. It was higher for social media posts that were actually true than for those that were actually false (χ^2^(1, 103) = 38.3, *p* < .001). With both *perceived accuracy* and *veracity* being coded as binary variables [1,0], the coefficient for *veracity* was 0.52 (SE = 0.05). *Perceived accuracy* did not vary by condition (χ^2^(1, 103) = 0.51, *p* = .47). The coefficient for *condition* was -0.02 (SE = 0.03). Additionally, there was no evidence that participants were more likely to perceive as true social media posts that were actually false in the self-certification condition than in the accuracy prompt condition (χ^2^(1, 103) = 0.14, *p* = .71). In this case the coefficient for *condition* was 0.02 (SE = 0.05).

*Share rate* was the percentage of posts that were shared. It was less for social media posts that were actual false than for those that were actually true (χ^2^(1, 149) = 30.6, *p* < .001). With *share rate* coded as a binary variable [1,0], the coefficient for *veracity* was 0.35 (SE 0.04). Furthermore, the *share rate* varied as a function of condition (χ^2^(2, 149) = 16.3, *p* < .001), being the least for the self-censorship condition. The coefficient for the accuracy prompt condition versus the baseline condition was -0.03 (SE = 0.04) and for the self-certification condition versus the baseline condition it was -0.15 (SE = 0.04). Sharing discernment also varied across the conditions (χ^2^(2, 149) = 21.2, *p* < .001). Sharing discernment was not different between the baseline and accuracy prompt conditions (χ^2^(1, 99) = 1.88, *p* = .17) with a coefficient of 0.05 (SE = 0.04) but was different between the accuracy prompt and self-certification conditions (χ^2^(1, 103) = 10.6, *p* = .001) with a coefficient of 0.12 (SE = 0.04) and the baseline and self-certification conditions (χ^2^(1, 96) = 19.8, *p* < .001) with a coefficient of 0.17 (SE = 0.04).

#### Discussion

In contrast to previous findings [1], our data show that sharing discernment was not greater in the accuracy prompt condition than in the baseline condition. However, sharing discernment was greater in the self-certification condition than in either the baseline condition or the accuracy prompt condition. Likely, in the accuracy prompt condition participants shared social media posts that they had identified as false (as evidenced by the share rate being greater than the perceived accuracy in this condition), whereas they were prevented from doing this in the self-certification condition. These results suggest that self-certification increases sharing discernment more than accuracy prompts.

## Experiment 2: The limits of self-certification

Self-certification works because most participants are not willing to lie and indicate that a post that they believe is false is true, just so that they are allowed to share it. It is possible that participants may be more willing to lie, if they were more invested in the subject matter. In the previous experiment, participants were based in the US but were shown social media posts that focused on the conflict in Ukraine. It is possible that these may not have been the most engaging posts for this group of participants. We were concerned that had we presented participants with posts that were more engaging, then participants may have been more willing to indicate that posts they thought might be false were true, just so they could share them. If this were indeed to occur, then it is possible that sharing discernment would not be superior in the self-certification condition than in an otherwise identical condition that does not involve self-certification. One aim of Experiment 2 was to investigate this possibility.

A second concern with Experiment 1 is that participants were asked to rate the veracity of the posts before being asked to share them. This was done so as to make the experiment as similar as possible in structure to the accuracy prompt experiments of [1]. However, if self-certification were employed in real-life, one would presumably ask participants to certify the veracity of only those posts that they wish to share. It is possible that participants may be more likely to rate a post as true if they have already indicated that they wish to share it. Thus, for Experiment 2, we reversed the order of the questions, having participants indicated whether they would share a post before rating its perceived accuracy.

### Method

#### Participants

Participants were recruited via Mechanical Turk, using the same exclusion criteria as before. Additionally, nobody who had participated in any of the previous experiments (including the pre-test experiment) was allowed to participate in this experiment. Using the previous pre-screener, we recruited an equal number of Republican and Democrats. Other political affiliations were not recruited. Participants were compensated US$1 for their time and the experiment took approximately 5 minutes to complete. We excluded only those participants who didn’t complete the experiment. Out of the 400 participants who completed the study, 212 self-identified as female, 184 as male. The mean age was 38.7 years old (SD = 12.0 years). 200 participants self-identified as Republicans, 200 as Democrats. All participants gave informed consent, and the study was approved by the University of Melbourne Ethics Advisory Group (ID: 23317).

#### Materials

For this experiment, it was crucial that we had a collection of social media posts that participants would really want to share. We obtained these posts in the following manner. Utilising Mechanical Turk workers who were located in the USA, had previously passed a test designed to assess English proficiency and to screen out bots, we ran a very short pre-screener to ask them with which political party they most strongly identified. We then selected 100 of those participants who indicated that they were either Republicans or Democrats (i.e. we excluded “independents”, “other party”, “not political”, “not aligned”). We then showed these participants 80 social media posts that were focused on US political issues. Half were true and half were false. Half were designed to favour the Democratic party and half were designed to favour the Republican party. For each post, we asked each participant whether they felt the post favoured the Republican party or the Democratic party, and whether they would share it. Using this data, we selected the ten posts that people most wanted to share in each of the following four categories: posts that were actually true that the majority of participants felt favoured the Republican party, posts that were actually true that were thought to favour the Democratic party, post that were actually false that were thought to favour the Republican party and posts that were actually false that were thought to favour the Democratic party. We used these posts in the subsequent experiment. This experiment was conducted 3–4 March, 2023.

#### Procedure

As before, the experiment started with the participants being asked to state their age, gender, and political affiliation. It was then explained that they would participate in a simulated social media study where their task was to gain as many followers as possible by sharing news posts that their followers wished to see. We used the same six news sources as previously, and it was explained which were reliable and which were unreliable. As before, the posts that were actually true were always paired with the reliable news sources whereas the posts that were actually false were always paired with the unreliable news sources.

We employed a two-by-two factorial design, resulting in four conditions. In half the conditions, the posts were fact-checked. This meant that for each post the participant was informed whether the post was true. The fact-checking was 100% accurate. In half the conditions, self-certification occurred whereas in the remaining conditions, it did not.

In all conditions, participants were told that they would see twenty social media posts, one at a time, and that for each post they would need to indicate first whether they wished to share it and then whether they thought the post was true. In the two self-certification conditions (i.e. Conditions 2 and 4), participants were additionally informed that they would only be able to share those posts that they indicated were true. In the two fact-checking conditions (i.e. Conditions 3 and 4), participants were told that each post had been fact-checked and they would be told if it was true or not. As before, participants were able to view their current number of followers. Every time they successfully shared a post, regardless of whether the post was actually true, their number of followers increased by a random number drawn from a normal distribution with a mean of 100 and a standard deviation of 20. The experiment was conducted 15–27 March, 2023.

### Results

**Fig 3:**
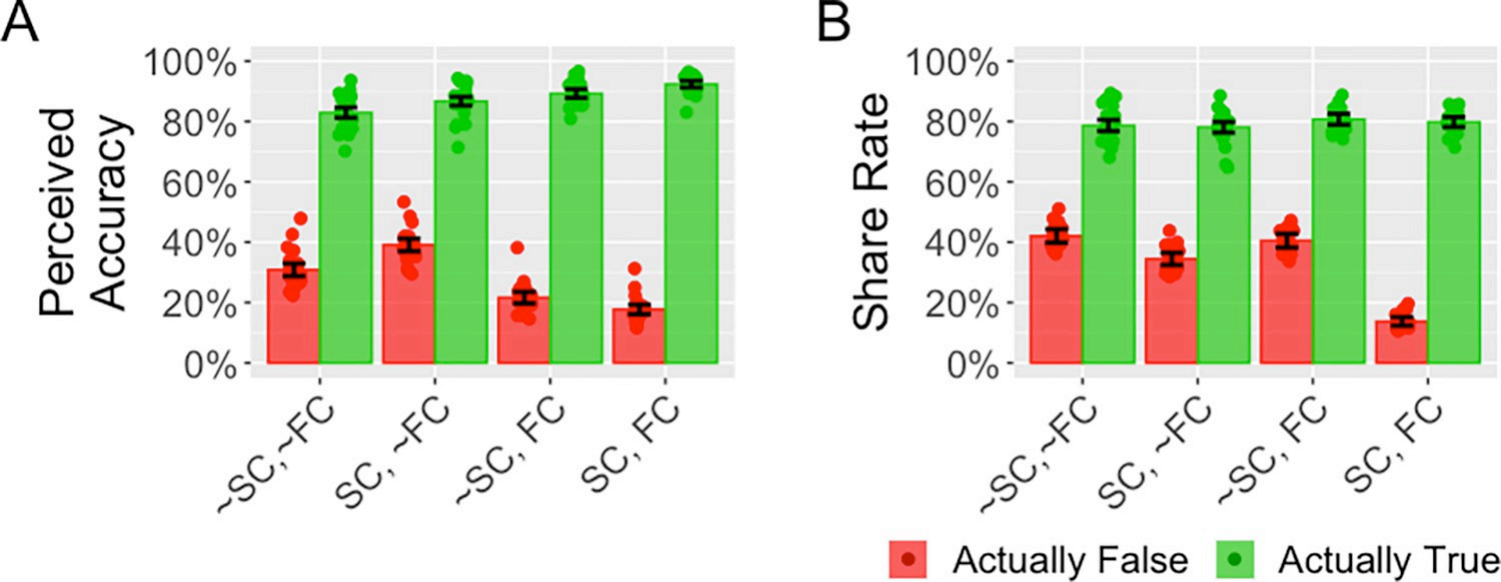
Results from Experiment 2. The conditions are described in Table 1. Subplot A shows the perceived accuracy, defined as the proportion of participants who indicated that each news post was true. Subplot B shows the share rate. Each dot shows the data averaged over a single social media post. The bars show the averages over all social media posts that were actually false (red) or actually true (green).

The results are shown in Fig 3. Subplot A shows the perceived accuracy. Considering only the social media posts that were actually false, there was an effect of *condition* on *perceived accuracy* (χ^2^(3,400) = 37.5, p < .001). Relative to the ~SC,~FC condition, the coefficient for the SC,~FC condition was 0.08 (SE = 0.04), the coefficient for the ~SC,FC condition was -0.09 (SE = 0.04), and the coefficient for the SC,FC condition was -0.13 (SE = 0.04). The *perceived accuracy* was lower in the two conditions with fact-checking (i.e. Conditions 3 and 4) than in the two conditions without fact-checking (i.e. Conditions 1 and 2) (χ^2^(1,400) = 32.0, p < .001) with the coefficient for *fact-checking* being -0.16 (SE = 0.03). But there was no effect of self-certification on perceived accuracy (χ^2^(1,400) = 0.35, p = .55) with the coefficient for *self-certification* being 0.02 (SE = 0.03). So, there was no evidence that participants were more likely to indicate that the actually false posts were true in the two self-certification conditions relative to the two conditions without self-certification.

**Table 1:** The four conditions used in Experiment 2. SC stands for self-certification, FC stands for fact-checking and “~” is used to denote the negation (i.e. absence) of either self-certification or fact-checking.

Condition	Description	Shorthand
**1**	No self-certification, no fact-checking	~SC, ~FC
**2**	Self-certification, no fact-checking	SC, ~FC
**3**	No self-certification, fact-checking	~SC, FC
**4**	Self-certification, fact-checking	SC, FC

Sharing discernment varied across conditions (χ^2^(3,400) = 353, p < .001). Relative to ~SC,~FC condition, the coefficient for the SC,~FC condition was 0.07 (SE = 0.02), the coefficient for the ~SC,FC condition was 0.04 (SE = 0.02), the coefficient for the SC,FC condition was 0.29 (SE = 0.02). There was an interaction between fact-checking and self-certification (χ^2^(4,400) = 361, p < .001), with a coefficient of -0.19 (SE = 0.03). Sharing discernment was greater in the SC,~FC condition than in the ~SC,~FC condition (χ^2^(1,199) = 14.2, p < .001) with a coefficient of 0.07 (SE = 0.02). It was not greater in the ~SC,FC condition than in the ~SC,~FC condition (χ^2^(1,183) = 3.56, p = .059) with a coefficient of 0.04 (SE = 0.02). As expected, sharing discernment was much greater in the SC,FC condition than in the ~SC,~FC condition (χ^2^(1,206) = 293, p < .001) with a coefficient of 0.29 (SE = 0.02). Sharing discernment was greater in the SC,FC condition than in the ~SC,FC condition (χ^2^(1,201) = 228, p < .001) with a coefficient of 0.26 (SE = 0.02). Overall, sharing discernment was greatest when there was both fact-checking and self-certification.

### Discussion

While fact-checking did have a large effect on the perceived accuracy of the social media posts that were actually false, it had no effect on sharing discernment unless it was paired with self-certification. Similarly, self-certification had only a small effect on sharing discernment unless it was paired with fact-checking. Therefore, for social media posts that participants are keen to share, it is necessary to combine self-certification with fact-checking. As shown by Condition 2, without fact-checking participants perceived almost 40% of the actually-false social media posts to be true, thereby rendering self-certification largely ineffective. Conversely, as shown by Condition 3, with fact-checking participants perceive only a small proportion of the actually-false social media posts to be true, but without self-certification they shared social media posts that they believe to be false. Fact-checking is needed to inform people which social media posts are likely to be false and self-certification is needed to ensure that participants don’t share posts they believe are false. Thus, both fact-checking and self-certification are needed to substantially increase sharing discernment.

## Experiment 3: Self-certification with crowd-based fact-checking

The previous experiment suggested that for self-certification to be effective, it needs to be accompanied by fact-checking so that people are made aware of which posts are likely to be false. However, it is unrealistic to expect experts to fact-check all relevant posts as there are too many of them to be fact-checked. As an alternative to expert fact-checking, we investigated whether crowd-based fact-checking is sufficient [30, 31]. The advantage of crowd-based fact-checking is that it can be done much more quickly. A potential disadvantage is that users may be less inclined to believe the opinion of the crowd. If so, crowd-based fact-checking might influence behaviour less than expert fact-checking. The purpose of this experiment was to investigate whether self-certification is effective when combined with crowd-based fact-checking.

As secondary aim was to compare self-certification with crowd-based fact-checking, henceforth referred to as “crowd self-certification” to accuracy nudges and media literacy training [11]. Experiment 1 utilised accuracy nudges, but that condition was not realistic as participants were required to judge the accuracy of every news post, something they typically not do on social media. Instead, adopting the procedure of [1], in the present experiment we had participants judge the accuracy of a single, unrelated social media news post before deciding whether or not share a series of other news posts. Such nudges could be implemented on social media. Pennycook et al. has previously shown that such accuracy nudges are effective at increasing sharing discernment [1].

Another common technique for increasing sharing discernment is via media literacy training [11]. We therefore devised a short medial literacy training program, tailored for the specific news posts used in our experiment. This training focused on three cues that would help participants determine whether the news post was likely to be accurate. Participants were then shown a series of news posts and decided which ones to share.

Based on our previous findings we expected that sharing discernment would not be greater in the accuracy nudge condition than in the baseline (i.e. control condition). However, we expected that sharing discernment would be greater in both the media literacy condition and in the crowd self-certification condition than in the baseline condition. We expected sharing discernment to be greater in the crowd self-certification condition than in the media literacy condition.

### Method

#### Participants

Participants were recruited via Mechanical Turk, using the same exclusion criteria as before. Additionally, nobody who had participated in any of the previous experiments (including the pre-test experiment) was allowed to participate in this experiment. Using the previous pre-screener, we recruited an equal number of Republican and Democrats. Other political affiliations were not recruited. Participants were compensated US$1 for their time and the experiment took less than 12 minutes to complete. We excluded only those participants who didn’t complete the experiment. Out of the 434 participants who completed the study, 185 self-identified as female, 248 as male and one as non-binary. The mean age was 36.9 years old (SD = 11.5 years). 230 participants self-identified as Republicans, 204 as Democrats. All participants gave informed consent, and the study was approved by the University of Melbourne Ethics Advisory Group (ID: 23317).

#### Materials

We used the same news posts as before, paired with the same six news sources as previously.

#### Procedure

As before, the experiment started with the participants being asked to state their age, gender, and political affiliation. It was then explained that they would participate in a simulated social media study where their task was to gain as many followers as possible by sharing news posts that their followers wished to see. Unlike before, we only explained which news sources were reliable and which were unreliable in the media literacy condition to ensure that this condition was distinct from the other conditions. Each participant was randomly assigned to one of the four conditions.

In the baseline condition (BL condition) participants then viewed the 40 news posts, one at time. For each news post they decide whether to share it. After making this decision, they were then shown their current number of followers before being shown the next news post. The accuracy prompt condition (AP condition) was identical to this condition except that participants first judge the accuracy of unrelated news post that focused on a topic not connected with USA politics. This was a social media post concerning Australia sourced from The New York Times claiming that since October 2019 it is illegal to climb Uluru (formerly known as Ayers Rock) due to it religious significance to the Pitjantjatjara people. The fine for doing so is up to AU$10,000. This post was completely true.

In the media literacy condition (ML condition) participants were given three tips to help determine whether a news post was accurate or not: the source of the news post, the tone of the post and whether the post cites any evidence to support its assertions. These tips were inspired by the tips shared in previous media literacy training experiments [11] but were specifically chosen to be highly reliable in the context of our experiment. It was explained that the logo of the source of each news post would appear under the post’s photograph. It was explained which of the three sources are reliable (The New York Times, The Wall Street Journal and the BBC) and which were unreliable (The Daily Buzz, Now8News and The World News Briefing). The logos of each source were shown to participants. Participants were then shown two posts in succession and asked to determine which was more likely to be inaccurate, based only on the sources. Immediately feedback was given.

It was then explained that the posts with a factual tone were more likely to be accurate than posts with a partisan tone. Two posts were then shown in succession and participants were asked to determine which was more likely to be inaccurate, based purely on the tone of the post. As before, immediate feedback was given.

It was then explained that posts that cite evidence to support their assertions are more likely to be accurate than those that do not. As before, to example posts were shown and participants were asked to determine which was more likely to be inaccurate, utilising only this cue. Immediate feedback was given.

Finally, two more posts were shown. For each post, participants need to determine whether it was likely to be accurate or inaccurate, and were then given immediate feedback. The first post was inaccurate. This post utilised an unreliable source, a partisan tone and did not cite any evidence to support its assertions. The second post was accurate. It utilised a reliable source, a factual tone and cited evidence to support its assertions.

As with the previously condition, participants were then shown 40 news posts, and for each news post needed to decide whether to share it or not. After each decision they were told their current number of followers. These were the same news posts as used in the other two conditions. The inaccurate news posts were always paired with one of the three unreliable news sources. Conversely, the accurate news posts were always paired with a reliable news post. The inaccurate news posts typically had a partisan tone and typically did not cite evidence to support their claims whereas the accurate news posts had a factual tone and typically cited evidence to support their assertions.

Using the data from the no self-certification, no fact-checking condition of Experiment 2, we calculated what proportion of the participants thought each post was true. For all the posts that were actually true, the majority of the participants thought the post was true. Similarly, for all the posts that were actually false, the majority of the participants thought the post was false. We used these findings as the basis the self-certification condition that used crowd-sourced fact-checking. This condition was identical to baseline condition except that every time a participant attempted to share a post that the crowd thought was false (i.e. an actually-false post) the participant was informed that the majority of previous participants thought that the post was false, so they could only share post if they believed it was accurate. They were then asked to indicate whether they thought it was accurate. Only if they indicated that they believed it was, was the post shared with their followers. We refer to this condition as the crowd self-certification condition (CSC condition).

As with the previous experiments, regardless of the condition and regardless of whether the post was actually true or not, every time a post was shared the participant gained a random number of new followers, drawn from a normal distribution with a mean of 100 and a standard deviation of 20. The experiment was conducted 15–27 March, 2023.

### Results

**Fig 4:**
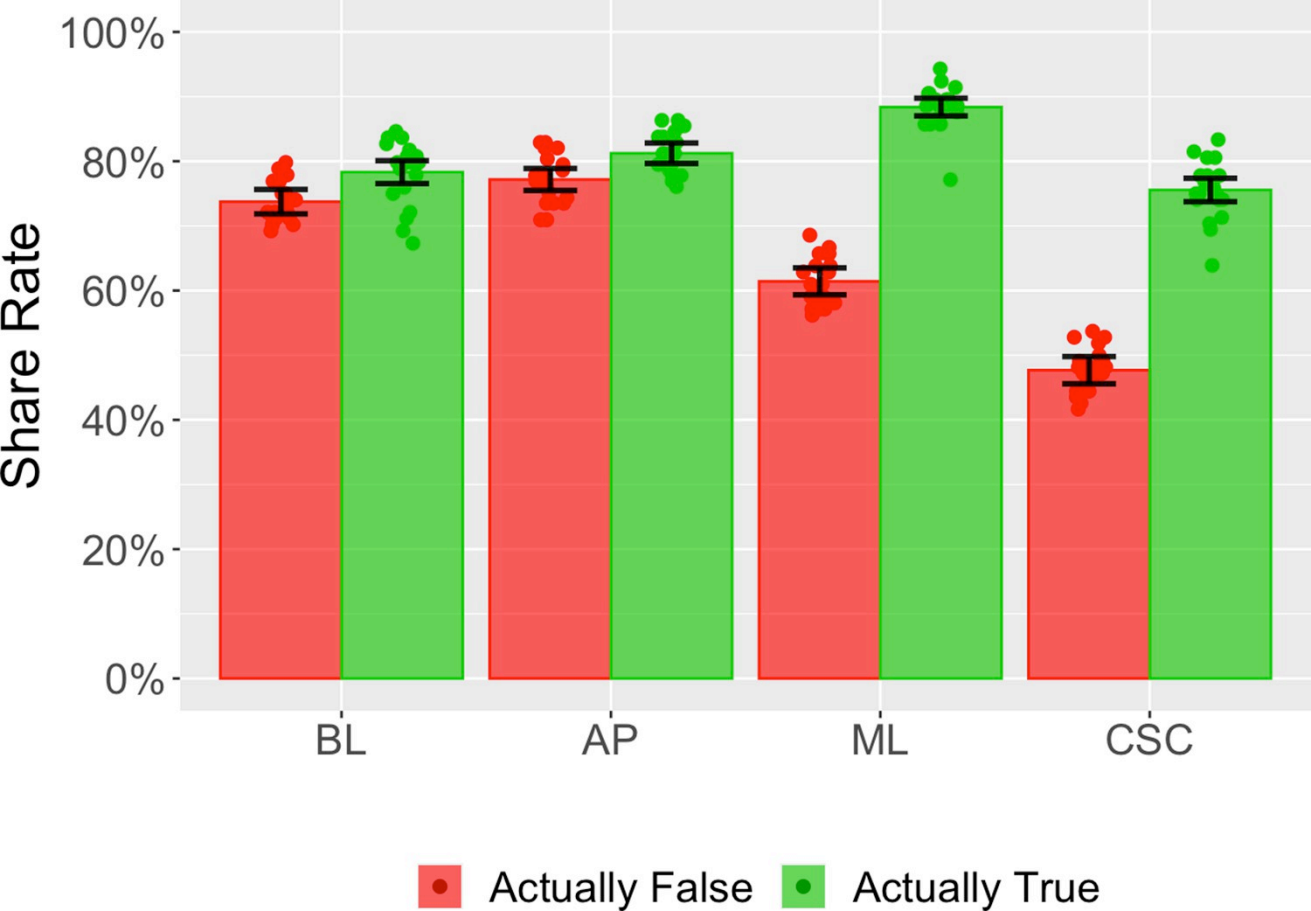
Results from Experiment 3. The conditions are described in Table 1. Each dot shows the data averaged over a single social media post. The bars show the averages over all social media posts that were actually false (red) or actually true (green). The four conditions were baseline (BL), accuracy prompt (AP), media literacy (ML) and crowd self-certification (CSC).

The results are shown in Fig 4. Sharing discernment varied across conditions, being greatest in the crowd self-certification condition (χ^2^(3,434) = 390, p < .001). Sharing discernment was not greater in accuracy prompt condition than in the baseline condition (χ^2^(1,221) = 0.1, p = .75, coefficient = -0.01, SE = 0.02) but it was greater in both the media literacy condition (χ^2^(1,209) = 182, p < .001, coefficient = 0.22, SE = 0.02) and in the crowd self-certification condition (χ^2^(1,212) = 177, p < .001, coefficient = 0.23, SE = 0.02) than in the baseline condition. Compared to the accuracy prompt condition, it was greater in the media literacy condition (χ^2^(1,222) = 214, p < .001, coefficient = 0.23, SE = 0.02) and in the crowd self-certification condition (χ^2^(1,225) = 207, p < .001, coefficient = 0.24, SE = 0.02). It was not greater in the crowd self-certification condition than in the media literacy condition (χ^2^(1,213) = 0.29, p = .59, coefficient = 0.01, SE = 0.02).

## General discussion

Most Americans now agree that steps should be taken to reduce the amount of online false information even if doing this limits freedom of speech [8]. However, there is no consensus on how this should be done. Two of the most popular methods are media literacy [11] and fact-checking [30, 32]. The problem with the former is that it requires extensive training [11], it is not clear how long the effects last for [33] and its efficacy is disputed [34, 35]. The problem with the latter is that the facts can often be disputed, with different fact checkers disagreeing as to the truthfulness of the statements that they fact check [36, 37]. As such, social media companies do not want to act as the ‘arbiters of the truth’ and would prefer not to censor posts based purely on fact checks [38].

To address this concern, we proposed that people should only be allowed to share posts that they have indicated are accurate. We referred to this intervention as self-certification. Across three experiments, we found that self-certification was highly effective. Experiments 1 and 3 showed that self-certification increased sharing discernment more than accuracy prompts and Experiment 3 showed that it increased sharing discernment as much as media literacy training, without requiring the training and retraining necessary for the media literacy intervention to work. Given that it can be hard to persuade people to be trained and periodically retrained, we would argue that self-certification is a more practical intervention.

We were surprised that accuracy prompts were not more effective. In neither Experiment 1 nor Experiment 3 were we able to show that they improved sharing discernment relative to the baseline condition. The effectiveness of accuracy prompts has previously been disputed in the literature [16, 17]. It appears that the effect size is relatively small and varies between studies [18]. This meta-analysis reported that the average effect size, as measured by the coefficient of the interaction between condition and veracity in a linear model, was 0.04. Considering only the baseline and the accuracy prompt conditions from Experiment 1 and Experiment 3, we found an interaction coefficient of 0.05 and -0.01 respectively. For comparison, we found an interaction coefficient of 0.17 and 0.23 respectively when considering only the baseline and the self-certification conditions. Self-certification is more effective than accuracy prompts.

We expected that some participants in the self-certification condition would lie and indicate that some of the news posts that they thought were false were true so that they would then be allowed to share them. There is no evidence that this occurred in Experiment 1 or in Experiment 2. In Experiment 1, the proportion of false news posts rated as true was not higher in the self-certification condition than in the accuracy prompt condition. Similarly, in Experiment 2, self-certification did not predict an increase in the proportion of actually-false posts perceived to be true. However, fact-checking was associated with perceptions of truth. In Experiment 2, for the posts that were actually false, fact-checking decreased the perceived accuracy. While there is no evidence that participants would lie and report as true that posts that they thought were false, there was evidence that, without fact-checking, about 40% of the posts that were actually false were perceived to be true. This proportion dropped to about 20% with fact-checking.

We were surprised by this result as we had taken efforts to draw our participants’ attention to the sources of the social media posts in that experiment. All social medial posts that were actually false were associate with an unreliable news source. Why then did they still perceive so many of these (actually-false) social media posts to be true? A possible reason is that even unreliable sources often report true news. It is not that unreliable sources go out of their way to only report falsehoods, rather they don’t employ the quality control procedures of mainstream news sources, so often report inaccurate information. This illustrates the limitations of source labelling. It can indicate what *might* be false but can’t indicate what *is* false.

This reinforces the need for fact-checking. However, our experiments showed us that fact-checking is not enough. In both Experiment 1 and in Experiment 2, participants repeatedly showed that they were willing to share posts they perceived to be false. As shown by Experiment 2, fact-checking on its own was ineffective: sharing discernment was not greater in Condition 3 than in Condition 1. To improve sharing discernment, one needs both fact-checking and self-certification.

When designing an intervention, one needs to balance the degree to which it restricts freedom of speech with the degree to which it is likely to reduce the propagation of misinformation. Self-certification restricts freedom of speech in that people are prevented from sharing social media posts that they have indicated are false. To what degree people should be allowed to knowingly spread false information is debated [39]. The concern is that if “lies” are not protected, an authoritarian government could silence its critics by accusing them of lying. Self-certification addresses this concern by devolving the decision as to whether the social media post is true or not to the user. This means that the user is the one to decide what is true so cannot be silenced by an authoritarian regime designating posts as false to prevent them being shared. In this way, self-certification protects democracy from authoritarianism.

Of course, the weakness of self-certification is that it relies on the user knowing which posts are false. As shown by Experiment 2, if it is unclear which posts are false, it may be necessary to combined it with fact-checking. Because people are willing to share posts they know to be false, fact-checking on its own is not always effective. However, the combination of fact-checking and self-certification is highly effective. As shown by Experiment 3, this combination of fact-checking and self-certification continues to be effective, even when the fact-checking is performed by the crowd. As it is unrealistic to expect experts to fact check all social media posts, we recommend that self-certification always be combined with crowd-based fact-checking.

This stance is somewhat different from our previous claim that self-certification on its own is sufficient to improve sharing discernment [17]. This previous work considered a situation where participants were less likely to perceive actually-false posts as true, thereby having less need for fact-checking to correct their misperceptions. Conversely, in Experiment 2, without fact-checking too many actually-false posts were perceived to be true for self-certification to improve sharing discernment.

Like all studies, ours had several limitations that would need to be rectified in future investigations. The most important limitation was that the experiments were done in a simulated setting. The participants were not actually sharing social media posts on an actual social media platform, rather they were on a simulated social media platform and not communicating with other participants. In future investigations, these studies would need to be performed on an actual social media platform with participants sharing posts with other people.

A second limitation is that in our experiments we informed our participants that their task was to gain followers by sharing social media posts. Arguably not all users of social media are trying to gain followers, so instructing our participants to behave in this way may have caused their behaviour to be unnatural. We told participants to do this because in most online communities most users are “lurkers” who hardly ever post or repost, and a minority create or repost most of the content [40, 41]. Obviously, misinformation is propagated only by those social media users who actual post or repost content. If we were to ask people to behave on the simulated platform in the same way that they behave on real social media platforms, most wouldn’t have shared anything. We therefore needed to explicitly encourage people to share. For the 1% of social media users who produce the most content, gaining followers is likely to be an important motivator. We therefore instructed our participants to attempt to gain followers because we wished them to emulate the behaviour of those people who were mostly likely to propagate misinformation. In this respect we were successful, with most of our users regularly sharing false information, which allowed us to study interventions to reduce the propagation of false information on social media.

A third limitation is that in our first two experiments (but not in the third) we informed participants which news sources were reliable and which were not. Conversely, in real life people would need to figure this out for themselves. We were therefore making the task easier than it would be in real life. We did this because our analysis required us be sure that participants knew which posts were likely to be false. Despite giving participants this information, many still shared posts that they believed to be false. So, this potential confound did not prevent us from replicating this phenomenon, which allowed us to compare different interventions for improving sharing discernment.

A fourth limitation is that we did not allow for the possibility that there may be legitimate reasons for sharing false posts. For instance, one might wish to share post to warn other users that the information is false. This is known as “pre-bunking” and is a useful way of reducing the spread of misinformation [14, 42]. Future studies will need to allow participants to share posts that they indicate are false with such posts being clearly indicated as false to the recipients.

A final limitation is that we prevented participants from revisiting their earlier decisions. For example, in Experiment 2, participants decided whether they wished to share a post before reflecting on its accuracy. It is possible that in the ~SC, FC condition if they decide that a post is false, they might wish to reverse their earlier decision to share it. Since in the SC, FC condition they would automatically be prevented from sharing such posts, which might inflate the sharing discernment of this condition relative to that of the previous condition. This difficulty is avoided by having participants decide whether a post is accurate before deciding whether to sharing it, which is what occurred in Experiment 1. Given both experiments found the same result, we can conclude that self-certification improves sharing discernment, regardless of the decision ordering.

In conclusion, this research has made a strong case for the utility of self-certification, especially when combined with crowd-based fact-checking. Consistent with previous findings, our research has shown that fact-checking on its own does not always improve sharing discernment. However, when combined with self-certification, it does. Given that fact-checking is expensive and time consuming, but self-certification is quick and cheap to implement, a strong case can be made that fact-checking should always be combined with self-certification. These two interventions complement each other as they address different cognitive blocks. Only combined do they provide an effective way of increasing sharing discernment.
